# Usefulness of a combination of an adaptive traction device and an insulated-type knife for a neoplastic lesion in ulcerative colitis

**DOI:** 10.1055/a-2240-9253

**Published:** 2024-02-02

**Authors:** Elena De Cristofaro, Jérôme Rivory, Louis-Jean Masgnaux, Jérémie Jacques, Timothée Wallenhorst, Pierre Lafeuille, Mathieu Pioche

**Affiliations:** 1 60259 Gastroenterology, University of Rome Tor Vergata Faculty of Medicine and Surgery, Rome, Italy; 2 36609 Gastroenterology and Endoscopy Unit, Hôpital Edouard Herriot, Lyon, France; 3 Hepatogastroenterology, CHU Dupuytren Limoges, Limoges, France; 4 36684 Department of Endoscopy and Gastroenterology, University Hospital Centre Rennes, Rennes, France

Endoscopic resection has been recommended by current guidelines for superficial colorectal neoplasia in patients with ulcerative colitis (UC), especially for clearly visible colitis-associated neoplasia 1 . Endoscopic submucosal dissection (ESD) is useful in such patients to ensure en bloc resection, minimizing the risk of recurrence and colectomy in the future; however, endoscopic removal is technically challenging in UC, mainly owing to underlying inflammation and fibrosis 2 . Several devices and techniques have been described to facilitate the procedure. The IT Knife nano (Olympus, Japan) is a knife with an insulated tip covering the needle and a metal disc at the back of the insulated tip. It is designed to be pulled for resection with a traction mechanism. Additionally, a traction strategy using adaptive traction (ATRACT) devices has already been described as being particularly useful in complex lesions 3 4 5 .

We report the case of 58-year-old patient with UC and a flat neoplastic lesion, 2 cm in size, in the sigmoid colon. The lesion was successfully resected thanks to the combined use of an ATRACT-2 and an IT Knife nano to optimize the traction force ( Video 1 ).

An adaptive traction device is used in combination with an insulated-type knife to resect a lesion in a patient with ulcerative colitis.Video 1

After a circumferential incision had been performed and trimmed with a DualKnife (Olympus, Japan), the two loops of the ATRACT-2 were fixed by clips to the edges of the lesion and the rubber band was fixed to the opposite colonic wall. After good submucosal exposure had been achieved, the dissection was started using the IT knife nano, which was moved from outside to inside with a pulling mechanism, with clear visualization of the lateral grooves ( Fig. 1 ). The procedure was completed in 18 minutes, without any adverse events. The resection was R0 and the histopathologic analysis revealed a nondysplastic serrated-like area.

**Fig. 1:**
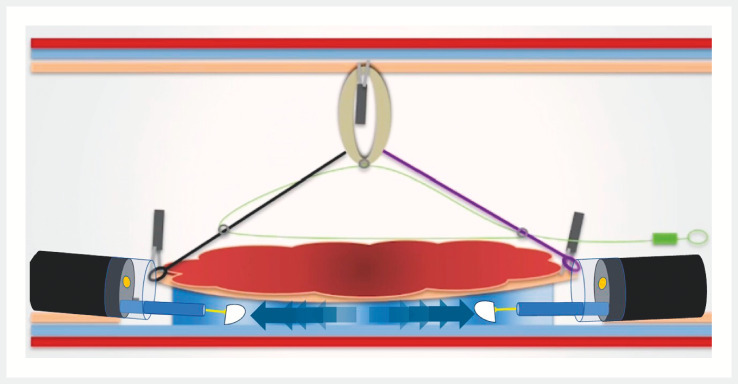
Schematic representation of the combined use of an ATRACT-2 and IT Knife nano in an endoscopic submucosal dissection procedure.

We hypothesize that such a combination of devices could make intervention safer and faster in selected patients, such as those with UC, in whom the technical difficulties may represent a great challenge.

Endoscopy_UCTN_Code_TTT_1AO_2AG

## Notes

Endoscopy E-Videos https://eref.thieme.de/e-videos
*E-Videos* is an open access online section of the journal *Endoscopy* , reporting on interesting cases and new techniques in gastroenterological endoscopy. All papers include a high-quality video and are published with a Creative Commons CC-BY license. Endoscopy E-Videos qualify for HINARI discounts and waivers and eligibility is automatically checked during the submission process. We grant 100% waivers to articles whose corresponding authors are based in Group A countries and 50% waivers to those who are based in Group B countries as classified by Research4Life (see: https://www.research4life.org/access/eligibility/ ). This section has its own submission website at https://mc.manuscriptcentral.com/e-videos .
